# Blood Cyst of the Mitral Valve Diagnosed in an Adult after Systemic Thrombolysis

**DOI:** 10.1155/2020/4320269

**Published:** 2020-07-27

**Authors:** Diana C. Ramírez-Mesías, Juan F. Contreras-Valero, Gabriel D. Pinilla-Monsalve, Carlos E. Vesga-Reyes

**Affiliations:** ^1^Hospital Universitario San Ignacio, Bogotá 110231, Colombia; ^2^Pontificia Universidad Javeriana, Bogotá 110231, Colombia; ^3^Fundación Valle del Lili, Cali 760032, Colombia; ^4^Universidad Icesi, Cali 760031, Colombia

## Abstract

Blood cysts in valves are very rare entities in adults, which can be distinguished through their echocardiographic features. A 57-year-old woman developed sudden dyspnea while hospitalized in the context of urinary sepsis; high-risk pulmonary embolism was diagnosed and she was prescribed systemic thrombolysis. She persisted with fever raising the suspicion of bacterial endocarditis. Transthoracic echocardiography did not report any masses, but later transesophageal imaging revealed a vegetation that was finally characterized as a blood cyst of the mitral valve based on ultrasound features. The patient evolved satisfactorily and did not require surgery.

## 1. Introduction

As detailed by Khan et al., intracardiac hematic cysts are benign “thin-walled structures lined by cobblestone-shaped epithelium, filled with non-organized blood” [1]. They were first defined by Elässer and are usually seen on atrioventricular valves in infants younger than six months but rarely in adults [2]. In 1983, Hausser et al. presented the first echocardiographic description of a hypoechogenic structure showing a synchronic movement with the tendinous cords towards the left ventricle outflow tract. Surgical and histological observations confirmed that this mass was consistent with a blood cyst [3]. Within the cardiac structure, they are mainly detected on valves with few cases of cysts attached to atria or ventricles [1]. Additionally, content echogenicity and intracystic microbubbles suggesting ventricle-to-cyst lumen connections [4] seem to be signs compatible with blood cysts.

Here, we report the case of a 57-year-old woman with a blood mitral valve cyst diagnosed after systemic thrombolysis for pulmonary embolism.

## 2. Case Presentation

This is the case of a 57-year-old Colombian obese woman who was admitted to the emergency department complaining of abdominal pain, urinary symptoms, and fever. Her medical history was positive for systemic arterial hypertension, type 2 diabetes mellitus, and nephrolithiasis. Ten days before the symptoms' onset, she underwent a dental procedure for tooth decay. In the beginning, she was treated in another facility for ketoacidosis, renal failure, and bacterial urinary tract infection, developing pneumonia as well. She required renal replacement therapy and received a course of antibiotics including aminopenicillins, carbapenems, and glycopeptides.

During hospitalization, the patient suddenly presented right calf pain; a duplex ultrasound was requested evidencing deep venous thrombosis of soleal and peroneal veins. She complained as well of dyspnea, chest pain, and palpitations, raising the suspicion of pulmonary embolism (Wells' criteria: 4.5 points). Thoracic CT angiography showed thrombosis of the pulmonary trunk and both main branches, and the initial transthoracic echocardiography (4 days after admission) reported left ventricle diastolic dysfunction with impaired relaxation, trivial mitral regurgitation, mild right ventricle dilation, mild tricuspid regurgitation, and an intermediate probability of pulmonary hypertension (PSAP 40 mmHg) with no other findings. Systemic thrombolysis with streptokinase was the chosen treatment for the high-risk pulmonary embolism; subsequently, she was started on enoxaparin with bridge therapy to warfarin.

After thrombolysis, the patient remained febrile with chest discomfort despite antibiotic treatment suggesting dialysis catheter-related infection or bacterial endocarditis. A transesophageal echocardiogram was carried out, revealing a mitral valve mass considered as a bacterial vegetation, and therefore, she continued receiving vancomycin. For exploring therapeutic alternatives, she was transferred to our hospital and stayed in the intensive care unit for one month. Her vital signs were normal (36.8°C, 95 beats per min, 20 breaths per min, 132/55 mmHg, SpO_2_ 94% on room air), but she was pale and cardiopulmonary auscultation was positive for S3 sound. No heart murmurs, abnormal breath sounds, or jugular ingurgitation was detected.

Laboratory analysis demonstrated leukocytosis (13.010 cells/*μ*L), moderate anemia (7.4 g/dL), mild thrombocytopenia (146.000/*μ*L), and increased C-reactive protein levels (17 mg/L, normal concentrations up to 5 mg/L). Moreover, renal function was severely compromised (creatinine 5.3 mg/dL and blood uremic nitrogen 42 mg/dL) and arterial blood gases showed metabolic acidosis (pH of 7.37, HCO_3_ 13.9 mmol/L, PCO_2_ 24 mmHg, SaO_2_ 87%, and base excess -10.6). Urine cultures were positive for IRT-resistant *Escherichia coli* and azole-sensitive *Candida albicans*, while no germs could be isolated on blood cultures. Consequently, fluconazole was added to the antibiotic regimen.

**Figure 1:**
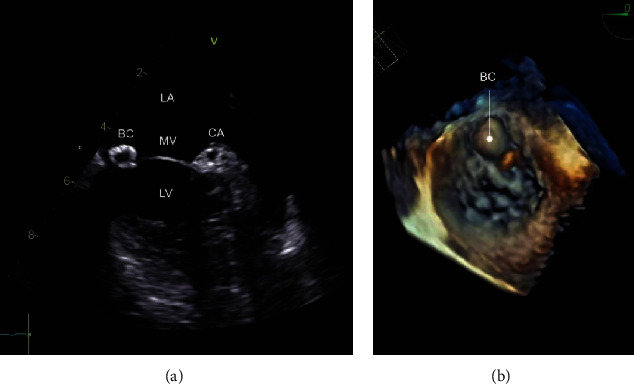
Transesophageal echocardiogram (a) with 3D reconstruction (b), exhibiting a blood cyst attached to the anterior leaflet of the mitral valve. LA: left atrium; LV: left ventricle; MV: mitral valve; BC: blood cyst; CA: circumflex artery.

Transesophageal echocardiogram (16 days after admission) demonstrated preserved left ventricular dimensions and ejection fraction. Mitral valve exhibited a rounded image of 10 × 10 mm with hyperrefringent edges and hypoechogenic content within the anterior ring, corresponding to A1 and A2 segments (Figure 1). This lesion did not compromise the valve function nor obstructed the left ventricle outflow tract. The rest of the valves, cavities, and vessels were normal, except for a small left pleural effusion. The density of the mass and the absence of any other imaging features compatible with endocarditis (i.e., highly and asynchronously mobile pedicled mass) supported the diagnosis of a blood cyst. Contrast echocardiogram with sulfur hexafluoride microbubbles was not available in our institution.

Aside from the mass, she did not fulfill any other Duke's criteria and blood cultures were negative. Besides, there was no valvular regurgitation that supported the diagnosis of a leaflet aneurysm and the cyst remained the same size in transesophageal echocardiogram despite anticoagulation, being less likely that it corresponded to a thrombus adhered to the valve in a patient with no predisposing factors. Researchers have highlighted elsewhere that echogenicity is the key to differentiate other entities (infections, neoplasms, and thrombi) from blood cysts as the last exhibit a homogenous hypoechogenic content [1]. Unfortunately, we could not obtain the initial transthoracic echocardiogram for reviewing and confirming that the mass was not present at that point.

After 10 days of antifungals, the inflammatory response completely resolved and the antibiotic course was not extended as bacterial endocarditis was disregarded. The patient did not present any bleeding or embolic events and was discharged without any disability. Unfortunately, she did not attend to the Outpatient Clinic but, in a telephone follow-up, no recurrence of cardiovascular symptoms was reported.

## 3. Discussion

Valvular blood cysts may be present in approximately 50% of random fetal and infant autopsies with diameters reaching up to 3 mm and a slight, but not significant, predominance in males (*p* = 0.103) [5]. In adults, cysts can be the consequence of blunt trauma and valvular surgery [6]. Until 2009, eleven cases of blood cysts on the mitral valve had been reported (54.54% were women, median age was 44 years, IQR 32-48) with the anterior leaflet (81.8%) as the most commonly affected structure [6]. Intracardiac cysts have been related to a variety of symptoms including dyspnea, chest pain at rest and on exertion [7, 8], or even syncope [9]; neurological deficits are less prevalent and associated with embolic stroke [1] or transient ischemic attack [3] after cyst rupture.

According to Dencker et al. and other authors, there are five hypotheses for explaining the occurrence of blood cysts: entrapment of blood during valve development; vascular occlusion in the setting of inflammatory, autonomic, metabolic, or hemorrhagic conditions generating subvalvular hematomas; presence of heteroplastic tissue from the embryonic pericardium; valvular blood vessel ectasia; and tumor proliferation in the form of angiomas [6]. We believe that in this patient there is a considerable probability that septic status due to urinary infection and systemic thrombolysis could have produced a hematoma in the shape of a cyst as proposed by the second hypothesis. Although the improbable non-regression of a congenital cyst cannot be excluded, these tend to be smaller [5] than those found in adults [6]. Furthermore, the lesion was not seen in the initial transthoracic echocardiogram and the patient's condition significantly improved without interventions other than stated, while no pathological sample could be obtained to favor different explanations. Histologically, cysts are delimited by homogeneous and dense connective tissue with layers of endothelial cells [5, 7] and occasional lymphocytic infiltrate [10].

Echocardiography is considered the method of choice for diagnosis [8] due to its accessibility and live assessment of valve function. However, cysts could be misdiagnosed as adherent thrombus [7] or bacterial vegetation such as in this case. Contrast echocardiography might be of help as stated before [4]. Related to other imaging modalities, cysts can be isodense in CT scans [8]; cardiac magnetic resonance (CMR) is usually obtained to clarify tissue characteristics, but blood cysts are variably observed as hyperintense in T2W [11], isointense in turbo spin echo [8], and/or with a prolonged T1 relaxation time in special breath-held inversion-recovery sequences [12]. Early gadolinium enhancement is not common [8].

For this patient, the blood cyst could have been overlooked in the initial transthoracic echocardiogram. In fact, limited sensitivity and negative likelihood ratio of 71% (95% CI 0.56-0.82) and 0.37 (95% CI 0.20-0.68), respectively, have been reported for detecting vegetations through this modality [13]. Furthermore, no diagnostic accuracy studies have been published comparing CMR with transesophageal echocardiography for identifying intracardiac blood cysts, but the last (with or without 3D reconstruction) has a discriminative capacity higher than 90% for thrombus or myxoma diagnosis. Transesophageal echocardiogram seems more accurate than CT scan, but false negatives can occur when masses are located in the left ventricle due to difficulty for visualizing the ventricular apex [14]. In the compilation of cases by Dencker, only 2 out of 11 cases had a cardiac MRI for supporting the diagnosis [6]; nevertheless, this modality has been relevant for confirming (rather than discarding) the echocardiographic finding [1, 8, 15]. Lamentably, there are still administrative barriers that impede access to advance image modalities in developing countries.

Currently, there are no treatment guidelines for blood cysts because of the low incidence, but the majority of symptomatic patients have undergone surgery for ruling out malignancy and preventing stroke [16]. *β*-blocking, antiaggregation, or anticoagulation remain controversial [15]. The prognosis is benign as congenital cysts tend to resolve spontaneously, but in fewer cases, valvular disease or obstruction of the ventricular outflow tracts can be present [9].

## 4. Conclusion

Intracardiac blood cysts are infrequent in adults and can be congenital or acquired after trauma or surgery. We presented the rare case of a blood cyst of the mitral valve in the context of severe infection and bleeding diathesis precipitated by thrombolysis. Echocardiographic assessment is useful for differential diagnosis based on functional and structural features. Treatment should be individualized according to clinical status and complications.

## Data Availability

Non applicable.
